# OA15.04. Accelerating the healing of bone fracture using homeopathy: a prospective, randomized double-blind controlled study

**DOI:** 10.1186/1472-6882-12-S1-O61

**Published:** 2012-06-12

**Authors:** S Sharma, N Sharma, R Sharma

**Affiliations:** 1 NMP Medical Research Institute, Jaipur, India; 2 Brett Research (UK), London, UK

## Purpose

In clinical practice, homeopathy is widely used in the fracture-repair process, which accelerates the healing of fractures, enhances callus formation and reduces pain. But there is no anatomical or scientific evidence yet to prove that. Therefore, the current study was undertaken to test the efficacy of homoeopathy in bone fracture healing.

## Methods

The study was conducted as a double blind randomized controlled study with 67patients with acute non-displaced lateral malleolar fracture. Patients were recruited from the Emergency Orthopaedic department, SMS Hospital, Jaipur, India during May 2007 to May 2009. Patients were randomized to either a homoeopathy treatment (n=34) or a control group (n=33). All the patients received standard orthopaedic care through 12 weeks following injury. The treatment group received homoeopathic medicine on the basis of totality of symptoms and individualisation. Outcome measures include radiological assessments and functional tests for healing. Assessments were taken on 3, 6, 9 and 12 weeks.

## Results

Faster healing was reported in the homeopathy group by week 9 following injury, including significant improvement in fracture line (p < 0.0001), fracture edge (p<0.0001), callous formation (p< 0.05) and fracture union (p< 0.0001) in comparison to placebo. There was also lower use of analgesics and less self-reported pain in the homeopathy group.

## Conclusion

The study suggests that homoeopathy could enhance anatomical and functional fracture healing.

